# Intraoperative hypotension is associated with shortened overall survival after lung cancer surgery

**DOI:** 10.1186/s12871-020-01062-2

**Published:** 2020-06-29

**Authors:** Wen-Wen Huang, Wen-Zhi Zhu, Dong-Liang Mu, Xin-Qiang Ji, Xue-Ying Li, Daqing Ma, Dong-Xin Wang

**Affiliations:** 1 Department of Anesthesiology and Critical Care Medicine, Peking University First Hospital, Beijing, 100034 China; 2 Department of Anesthesiology, Peking University Cancer Hospital, Beijing, 100083 China; 3 Department of Medical Records and Statistics, Peking University Cancer Hospital, Beijing, 100083 China; 4 Department of Biostatistics, Peking University First Hospital, Beijing, 100034 China; 5 Section of Anaesthetics, Pain Medicine and Intensive Care, Department of Surgery and Cancer, Imperial College London, London, SW10 9NH UK; 6 Department of Outcomes Research Consortium, Cleveland Clinic, Cleveland, OH USA

**Keywords:** Lung neoplasms, Thoracic surgical procedures, Hypotension, Prognosis

## Abstract

**Background:**

Intraoperative hypotension is associated with increased morbidity and mortality after surgery. We hypothesized that intraoperative hypotension might also be associated with worse long-term survival after cancer surgery. Herein, we analyzed the correlation between intraoperative hyper−/hypotension and overall survival after lung cancer surgery.

**Methods:**

In this retrospective cohort study, 676 patients who received lung cancer surgery between January 1, 2006 and December 31, 2009 were reviewed. Intraoperative hyper- and hypotension were defined according to their correlation with long-term survival. The primary endpoint was overall survival. The association between episodes of intraoperative hyper−/hypotension and overall survival was analyzed with multivariable Cox proportional hazard models.

**Results:**

Long-term follow-ups were completed in 515 patients with a median duration of 5.2 years. The estimated 5-year survival rates were 66.5, 61.3, 56.5, and 41.2% in patients with only hypertension (systolic blood pressure > 140 mmHg for ≥5 min), with both hyper- and hypotension (systolic blood pressure < 100 mmHg for ≥5 min), with neither hyper- nor hypotension, and with only hypotension during surgery, respectively. After adjusting confounding factors, intraoperative hypotension was significantly associated with shortened overall survival (compared with patients with only intraoperative hypertension, those with both hyper- and hypotension: hazard ratio [HR]1.033, 95% confidence interval [CI] 0.709 to 1.507, *p* = 0.864; those with neither hyper- nor hypotension: HR 0.952, 95% CI 0.608 to 1.489, *p* = 0.829; those with only hypotension: HR 1.736, 95% CI 1.218 to 2.475, *p* = 0.002).

**Conclusions:**

For patients undergoing lung cancer surgery, intraoperative hypotension, but not hypertension, was associated with shortened overall survival.

## Background

Lung cancer accounts for 13% of the total cancer diagnosis and is the primary cause of cancer deaths in males and the second cause of cancer death in females globally [1]. It has the highest incidence and mortality among all malignant tumors in China [2]. Timely surgery is the front-line therapy for lung cancer and hence, continuous efforts have been made to evolve surgical strategies and techniques [3, 4]. However, perioperative period is characterized with profound changes in immune function which are attributed by anesthesia- and surgery-related factors including volatile anesthetics and opioids, surgical trauma, bleeding and blood transfusion, hypothermia, neuroendocrine stress response, and inflammation. It has been suggested that anesthetic management may also affect long-term outcome of patients after cancer surgery [5–7].

Indeed, even when well-controlled, hypo- and hypertensive episodes are common during anesthesia and surgery; and poor management of intraoperative blood pressure was associated with the occurrence of perioperative cardiovascular events, organ injury and mortality [8–10] and even 1-year mortality [11]. In a cohort study of patients undergoing surgery for colorectal liver metastases, high number of intraoperative hypotensive episodes was associated with shortened recurrence-free survival [12]. On the other hand, it was reported that long-term survival was compromised by perioperative hypertension in renal or rectal cancer patients [13, 14]. We hypothesized that intraoperative hypo−/hypertension might also affect long-term survival after lung cancer surgery. However, evidences are lacking on this topic. This study aimed to analyze the possible association between intraoperative hyper−/hypotensive episodes and overall survival in patients after surgery for lung cancer.

## Methods

This retrospective cohort study was a further analysis of our patients’ data [15]. The study protocol received ethics approval from the Clinical Research Ethics Committee of Beijing University Cancer Hospital (2014[074]). Written informed consents from patients were waived by the Ethics Committee because the nature of study was pure observational, and no intervention was given to any patients; but all enrolled patients verbally agreed to participate in long-term follow-up. All the collected data were protected, and patient’s confidentiality was guaranteed.

### Patients

Consecutive patients who received intrathoracic lung surgery between January 1, 2006 and December 31, 2009 in Peking University Cancer Hospital were screened. The inclusion criteria included: (1) age ≥ 18 years, (2) lung cancer diagnosis was confirmed by pathological examination after surgery, and (3) the data of intraoperative blood pressure could be tracked in the electronic anesthesia record system. Patients who met any of the following criteria were excluded: (1) existence of primary cancer in other place, (2) metastatic or recurrent lung cancer, (3) lost to follow-up, and (4) manually recorded intraoperative monitoring data or missing data.

### Collection of baseline and perioperative data

Data collection was performed by qualified researchers from inpatient medical record system. Baseline data included age, sex, body mass index (BMI), smoking history, preoperative comorbidity, previous history of chemotherapy for cancer, and American Society of Anesthesiologists (ASA) physical status classification. Intraoperative data included methods of anesthesia, types and doses of anesthetics, estimated blood loss, infusion of blood products, uses and doses of glucocorticoids, uses and doses of nonsteroidal anti-inflammatory drugs, type and duration of surgery, and performance of mediastinal lymph node dissection. Postoperative data included pathological diagnosis, maximal tumor size, grade of tumor cell differentiation, pathological Tumor-Node-Metastasis (TNM) stage [16], and occurrence of complications during hospital stay after surgery.

### Data acquisition for intraoperative blood pressure

The readings of intraoperative blood pressure, which were stored in the electronic anesthesia record system and were recorded at least every 5 min during operation, were obtained from each patient’s electronic anesthesia chart. Blood pressure was monitored via a standard automatic arm cuff or through an intra-arterial catheter. When both non-invasive and invasive measurements were performed, invasive blood pressure was adopted for analysis. The last systolic blood pressure (SBP) reading before anesthesia induction was recorded as pre-anesthesia SBP. The time of anesthesia induction was defined as the moment of anesthetic administration or 3 min before the first reading of expired carbon dioxide, whichever came first [17].

Intraoperative hyper- and/or hypotension were defined according to the absolute thresholds and durations of SBP. We adopt this criterion because it is commonly used in daily practice than either relative thresholds (such as percent change from baseline) or mean blood pressure, and SBP is the primary target of intervention [18]. The criteria of intraoperative hypertension (SBP > 140 or 160 mmHg, for a minimal duration of 5 or 10 min) and hypotension (SBP < 100 or 90 mmHg, for a minimal duration of 5 or 10 min) were chosen based on the literature [9, 11, 19].

### Postoperative follow-up

Postoperative follow-up was performed with outpatient interview, telephone interview or letter communication. Patients were followed-up at 6 and 12 months during the first year after surgery, and then once a year thereafter. Data of postoperative treatment including chemo- and/or radiotherapy were collected. The status of survival was confirmed during each follow-up, and the date and causes of death (if occurred) were recorded according to the medical certificate of death. Follow-up was continued until the patient died or was lost to follow-up. For all cases, follow-up was conducted by trained staff from the Department of Medical Records and Statistics of Peking University Cancer Hospital. The primary endpoint was overall survival, i.e., the duration from surgery to death of any cause.

### Statistical analysis

Continuous data with non-normal distribution were reported as median (interquartile range [IQR]). Categorical data were reported as numbers (%). The association between baseline/perioperative variables and long-term overall survival were analyzed with univariate Cox proportional hazards regression analyses based on clinical importance and the literature [15, 20–23]. The criteria of absolute thresholds and episode durations for the diagnosis of intraoperative hypo−/hypertension were decided according to the hazard ratios estimated with Cox proportional hazard regression models with or without adjustment for confounding factors (set as p < 0.20 in univariable analyses or were considered as clinically important). Postoperative survival estimation was made by Kaplan-Meier survival analysis for patients with four combinations of intraoperative hypertension (yes, no) and intraoperative hypotension (yes, no). The association between different combinations of intraoperative hyper−/hypotension and overall survival were analyzed with Cox proportional hazard regression models and adjusted for confounding factors. Missing data were not replaced. All tests were two-sided. A two-sided p < 0.05 was considered to be statistically significant. Bonferroni correction was performed for multiple comparisons. The statistical software package SPSS version 25.0 (IBM SPSS Inc., Chicago, IL, USA) was used for all analyses.

Sample-size calculation was not performed beforehand. However, considering the high number of events (nearly 270 deaths) compared with the number of variables (18 variables) included in the Cox model, the “ten events per variable” rule was exceeded, indicating sufficient accuracy of the regression estimates [24].

## Results

### Patient recruitment and follow-up results

**Fig. 1:**
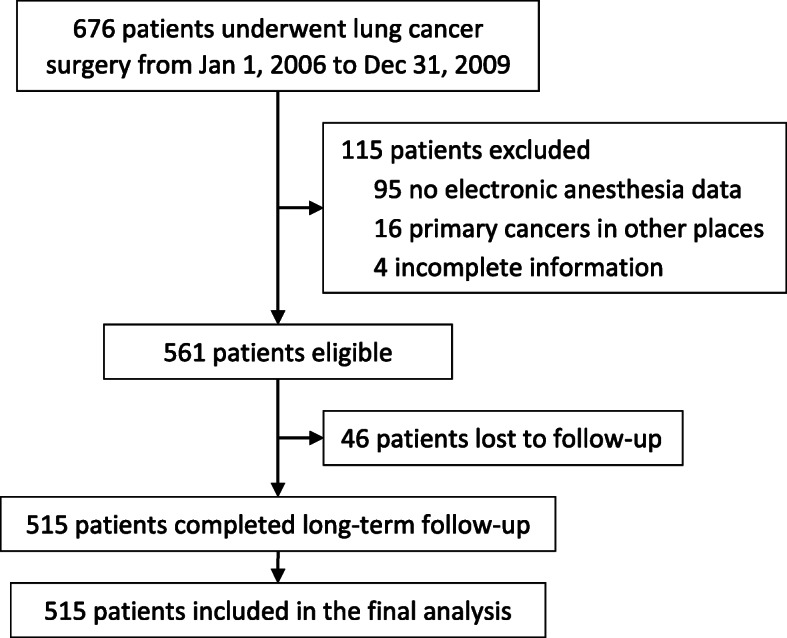
Flowchart of the study

Six hundred seventy-six patients underwent lung cancer surgery from January 1, 2006 to December 31, 2009. Of these, 561 met the eligibility criteria, 515 completed the long-term follow-ups and were included for the final analysis (Fig. 1). The last follow-up was performed on December 31, 2015. The median follow-up interval was 5.2 years (IQR 2.0–6.6). At the end of the long-term follow-up, 263 patients (51.1%) died and among them, 249 (94.7%) died of cancer. The median duration of overall survival was 63.2 months (IQR 28.1–79.5) (See Additional files 1 and 2).

### Potential confounding factors of overall survival

Eighteen factors were identified by univariable analyses (*p* < 0.20) or considered clinically important, including age, body mass index, male sex, chronic smoking, history of hypertension, preoperative chemotherapy, perioperative sufentanil equivalent, perioperative dexamethasone, perioperative flurbiprofen axetil, intraoperative blood transfusion, conservative resection/biopsy (vs. other types of surgery), mediastinal lymph node dissection, histological type as small-cell lung cancer, maximal tumor size, tumor differentiation, pathological Tumor-Node-Metastasis (TNM) stage, occurrence of postoperative complications, and postoperative chemo−/radiotherapy (Table 1). Of these, maximal tumor size was excluded from further multivariate analysis because it was closely related to pathological TNM stage.

**Table 1:** Baseline and perioperative variables and their univariate association with overall survival

Factors	Variables (*n* = 515)	Univariate HR (95% CI) ^a^	*p* value
Age (yr.)	61 (53–67)	1.201 (0.941–1.533)	0.141
Body mass index (kg m^− 2^)	24.2 (22.2–26.1)	0.956 (0.921–0.992)	0.018
Male sex	328 (63.7%)	1.473 (1.132–1.916)	0.004
Chronic smoking ^b^	277 (53.8%)	1.296 (1.015–1.656)	0.038
Preoperative comorbidity			
Coronary heart disease	30 (5.8%)	0.868 (0.507–1.487)	0.606
Hypertension	135 (26.2%)	0.644 (0.478–0.869)	0.004
Diabetes mellitus	56 (10.9%)	0.889 (0.597–1.324)	0.563
Stroke	17 (3.3%)	1.417 (0.775–2.592)	0.258
Preoperative chemotherapy^c^	57 (11.1%)	1.608 (1.136–2.278)	0.007
Charlson Comorbidity Index ^d^	0 (0–0)	1.010 (0.798–1.279)	0.934
ASA classification			
I	183 (35.5%)	1.000	
II + III	332 (64.5%)	0.851 (0.663–1.093)	0.205
Pre-anesthesia SBP (mmHg) ^e^			
< 120	85 (16.5%)	1.000	
120–139	153 (29.7%)	1.093 (0.758–1.576)	0.633
140–159	158 (30.7%)	0.808 (0.556–1.175)	0.264
≥ 160	119 (23.1%)	1.015 (0.690–1.492)	0.940
Combined epidural-general anesthesia (vs. general anesthesia)	110 (21.4%)	0.948 (0.707–1.272)	0.723
Use of general anesthetics			
Propofol (vs. no use)	459 (89.1%)	1.207 (0.799–1.823)	0.372
Etomidate (vs. no use)	118 (22.9%)	0.944 (0.814–1.094)	0.446
Nitrous oxide (vs. no use)	94 (18.3%)	0.951 (0.820–1.102)	0.504
Sevoflurane (vs. no use)	198 (38.4%)	0.944 (0.739–1.207)	0.647
Isoflurane (vs. no use)	310 (60.2%)	1.067 (0.835–1.365)	0.603
Perioperative sufentanil equivalent (μg)	280 (93–320)	0.999 (0.998–1.000)	0.033
Perioperative use of dexamethasone	266 (51.7%)	0.776 (0.608–0.989)	0.041
Perioperative use of flurbiprofen axetil	311 (60.4%)	0.761 (0.596–0.971)	0.028
Intraoperative crystalloid (ml)	1350 (1100–1600)	1.000 (1.000–1.000)	0.434
Intraoperative artificial colloid (ml)	500 (500–1000)	1.000 (1.000–1.000)	0.538
Intraoperative vasoactive drugs	101 (19.6%)	1.067 (0.793–1.437)	0.668
Intraoperative blood transfusion	9 (1.7%)	3.018 (1.490–6.109)	0.002
Duration of surgery (hr.)	4.0 (3.0–4.0)	0.952 (0.853–1.064)	0.386
Conservative resection or biopsy (vs. other types of surgery) ^f^	45 (8.9%)	2.995 (2.112–4.247)	< 0.001
Mediastinal lymph node dissection	461 (89.5%)	0.429 (0.307–0.599)	< 0.001
Histological type as small-cell lung cancer	16 (3.1%)	2.584 (1.477–4.521)	0.001
Maximal tumor size (cm)	3.0 (2.0–4.0)	1.175 (1.106–1.249)	< 0.001
Tumor differentiation			
Highly differentiated	62 (11.0%)	1.000	
Moderately differentiated	315 (61.2%)	2.047 (1.257–3.332)	0.004
Poorly differentiated	60 (11.7%)	3.671 (2.116–6.369)	< 0.001
Undifferentiated	78 (15.1%)	2.420 (1.390–4.214)	0.002
Pathological TNM stage ^g^			
I	201 (41.5%)	1.000	
II	100 (20.7%)	2.762 (1.879–4.060)	< 0.001
III	140 (28.9%)	4.388 (3.118–6.176)	< 0.001
IV	43 (8.9%)	6.781 (4.398–10.457)	< 0.001
Occurrence of postoperative complications ^h^	370 (71.8%)	1.387 (1.043–1.845)	0.024
Postoperative chemo−/radiotherapy	283 (55.0%)	1.531(1.198–1.957)	0.001

Data are median (interquartile range) or number (%)

*Abbreviations*: *HR* hazard ratio, *CI* confidence interval, *ASA* American Society of Anesthesiologists, *SBP* systolic blood pressure, *NSAIDs* non-steroidal anti-inflammatory drugs, *TNM stage* Tumor-Node-Metastasis stage

^a^ Performed with COX proportional-hazards regression analyses

^b^ Smoking of half a pack of cigarettes per day for at least 2 years, either former or current smoker

^c^ Missing data in 1 patient

^d^ According to the 1987 version without age correction

^e^ Defined as the last systolic blood pressure reading in the operating room before anesthesia induction

^f^ Performed in patients with nonresectable cancer; compared with lobectomy, pneumonectomy, wedge resection, and bronchial resection. Missing data in 7 patients

^g^ According to the 7th edition of the American Joint Committee on Cancer staging system. Missing data in 31 patients

^h^ Defined as newly occurred medical conditions that required therapeutic intervention during hospital stay after surgery (see supplement Table 2)

### Criteria of intraoperative hypo- and hypertension

Potential criteria included four thresholds of SBP (higher than 140 or 160 mmHg, and lower than 100 or 90 mmHg) with two minimal length of durations (5 or 10 min). Results of Cox proportional hazard regression analyses (Table 2) showed that intraoperative hypertension, defined as SBP > 140 mmHg for at least 5 min, was associated with prolonged overall survival (adjusted hazard ratio [HR] 0.719, 95% confidence interval [CI] 0.545 to 0.948, *p* = 0.019); whereas intraoperative hypotension, defined as SBP < 100 mmHg for at least 5 min, was associated with shortened overall survival (adjusted HR 1.382, 95% CI 1.047 to 1.825, *p* = 0.023). Therefore, SBP > 140 mmHg for at least 5 min was adopted as the criterion of intraoperative hypertension, and SBP < 100 mmHg for at least 5 min was adopted as the criterion of intraoperative hypotension in the subsequent analyses.

**Table 2:** Association between different threshold and duration of intraoperative hyper−/hypotension and overall survival

Thresholds	N	Episode duration of ≥5 min		N	Episode duration of ≥10 min	
		Unadjusted HR (95% CI) ^a^	Adjusted HR (95% CI) ^a,b^		Unadjusted HR (95% CI) ^a^	Adjusted HR (95% CI) ^a,b^
Intraoperative SBP > 160 mmHg	101	1.132 (0.841–1.532)	1.371 (0.974–1.929)	50	1.064 (0.714–1.584)	1.516 (0.980–2.343)
Intraoperative SBP > 140 mmHg	286	**0.671 (0.526–0.854)**	**0.719 (0.545–0.948)**	201	**0.687 (0.532–0.887)**	0.795 (0.594–1.063)
Intraoperative SBP < 100 mmHg	279	**1.371 (1.072–1.754)**	**1.382 (1.047–1.825)**	201	1.084 (0.846–1.387)	1.118 (0.840–1.488)
Intraoperative SBP < 90 mmHg	73	0.993 (0.701–1.406)	0.903 (0.613–1.330)	46	1.111 (0.736–1.679)	1.040 (0.652–1.660)

*Abbreviations*: *N* number of patients with events, *HR* hazard ratio, *CI* confidence interval, *SBP* systolic blood pressure. Results in bold indicate those with p < 0.05

^a^ Performed with COX proportional-hazards regression analyses

^b^ Adjusted for age, body mass index, male gender, chronic smoking, history of hypertension, preoperative chemotherapy, perioperative sufentanil equivalent, perioperative dexamethasone, perioperative flurbiprofen axetil, intraoperative blood transfusion, conservative resection/biopsy (vs. other types of surgery), mediastinal lymph node dissection, small cell lung cancer, tumor differentiation, pathological Tumor-Node-Metastasis stage, occurrence of postoperative complications, and postoperative chemo−/radiotherapy. Maximal tumor size was excluded due to correlation with pathological Tumor-Node-Metastasis stage

### Intraoperative blood pressure events and long-term survival

**Fig. 2:**
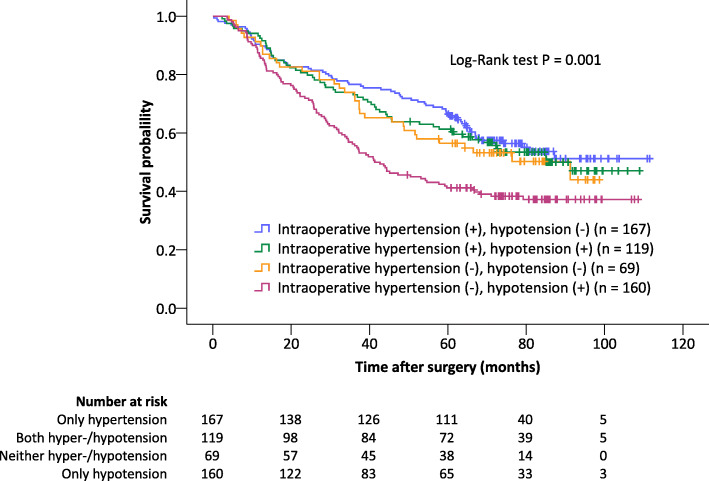
Survival curves of patients with 4 combinations of intraoperative hyper- and hypotensive episodes. Patients who experienced only hypotension during surgery had a significantly shortened overall survival than those who experienced only hypertension (*p* < 0.001) and those who experienced both hypertension and hypotension (*p* = 0.008) (adjusted significance criterion after Bonferroni correction was *p* < 0.0167). The cross signs indicate censored data

The estimated 5-year survival rates were 66.5, 61.3, 56.5, and 41.2% in patients with only hypertension, with both hyper- and hypotension, with neither hyper- nor hypotension, and with only hypotension during surgery, respectively (Table 3). Patients who experienced only hypotension during surgery had a significantly shortened overall survival when compared with those who experienced only hypertension (*p* < 0.001) and those who experienced both hyper- and hypotension (*p* = 0.008) during surgery (adjusted significance criterion after Bonferroni correction was *p* < 0.0167) (Fig. 2). After adjusting potential confounding factors, patients who experienced only intraoperative hypotension had a significantly shortened overall survival when compared with those who experienced only intraoperative hypertension (adjusted HR 1.736, 95% CI 1.218 to 2.475, *p* = 0.002) (Table 4).

**Table 3:** Estimated survival status of patients with different combinations of intraoperative hyper−/hypotension

Conditions	N	Mean overall survival in months (95% CI)	1-yr survival rate in % (95% CI) ^a^	3-yr survival rate in % (95% CI) ^a^	5-yr survival rate in % (95% CI) ^a^
Intraoperative hypertension (+), hypotension (−) ^b^	167	76.9 (70.6–83.2)	89.8 (85.3–94.3)	76.6 (70.1–83.1)	66.5 (59.2–73.8)
Intraoperative hypertension (+), hypotension (+) ^b^	119	72.1 (64.8–79.4)	92.4 (87.7–97.1)	73.1 (65.1–81.1)	61.3 (52.5–70.1)
Intraoperative hypertension (−), hypotension (−) ^b^	69	65.6 (57.1–74.1)	89.9 (82.8–97.0)	72.5 (61.9–83.1)	56.5 (44.7–68.3)
Intraoperative hypertension (−), hypotension (+) ^b^	160	58.3 (51.9–64.7)	85.6 (80.1–91.1)	55.6 (48.0–63.2)	41.2 (33.6–48.8)

Data are mean duration of overall survival in months or survival rate in % (95% CI)

*Abbreviations*: *N* number of patients with events, *CI* confidence interval

^a^ Estimated with Kaplan-Meier analyses

^b^ Intraoperative hypertension was defined as a systolic blood pressure > 140 mmHg for ≥5 min; intraoperative hypotension was defined as a systolic blood pressure < 100 mmHg for ≥5 min

**Table 4:** Association between different combinations of intraoperative hyper−/hypotension and duration of overall survival

Conditions	N	Unadjusted ^a^		Adjusted ^a,b^	
		Hazard ratio (95% CI)	p value	Hazard ratio (95% CI)	p value
Intraoperative hypertension (+), hypotension (−) ^c^	167	Ref.		Ref.	
Intraoperative hypertension (+), hypotension (+) ^c^	119	1.116 (0.789–1.579)	0.534	1.033 (0.709–1.507)	0.864
Intraoperative hypertension (−), hypotension (−) ^c^	69	1.198 (0.797–1.800)	0.384	0.952 (0.608–1.489)	0.829
Intraoperative hypertension (−), hypotension (+) ^c^	160	1.746 (1.290–2.364)	< 0.001	1.736 (1.218–2.475)	0.002

*Abbreviations*: *N* number of patients with events, *CI* confidence interval

^a^ Performed with COX proportional-hazards regression analyses

^b^ Adjusted for age, body mass index, male gender, chronic smoking, history of hypertension, preoperative chemotherapy, perioperative sufentanil equivalent, perioperative dexamethasone, perioperative flurbiprofen axetil, intraoperative blood transfusion, conservative resection/biopsy (vs. other types of surgery), mediastinal lymph node dissection, small cell lung cancer, tumor differentiation, pathological Tumor-Node-Metastasis stage, occurrence of postoperative complications, and postoperative chemo−/radiotherapy. Maximal tumor size was excluded due to correlation with pathological Tumor-Node-Metastasis stage

^c^ Intraoperative hypertension was defined as a systolic blood pressure > 140 mmHg for ≥5 min; intraoperative hypotension was defined as a systolic blood pressure < 100 mmHg for ≥5 min

## Discussion

In this retrospective cohort study, 515 patients were followed up for a median of 5.2 years after lung cancer surgery. Intraoperative hypertension (SBP > 140 mmHg for at least 5 min) and hypotension (SBP < 100 mmHg for at least 5 min) were defined according to their association with overall survival. When compared with patients who experienced only intraoperative hypertension, those who experienced only intraoperative hypotension had significantly shortened overall survival after correction for confounding factors.

The definition of intraoperative hyper−/hypotension varied widely in the literature. It is generally accepted that SBP > 160 mmHg during surgery is defined as intraoperative hypertension, and SBP < 80 mmHg as hypotension [9, 17]. In a recent consensus statement, it is suggested that brief durations of SBP < 100 mmHg are harmful during noncardiac surgery [25]. However, these definitions are usually introduced according to their effects on perioperative “short-term” outcomes. In the present study, we defined intraoperative hyper−/hypotension according to their effects on long-term survival. As such, we adopted an episode of SBP > 140 mmHg for at least 5 min as intraoperative hypertension and an episode of SBP < 100 mmHg for at least 5 min as hypotension.

Perioperative hypertension is associated with an increased risk of adverse events, including cardiovascular morbidity or even death [26, 27]. Regarding long-term outcome, it was reported that pre- and postoperative hypertension negatively affects long-term survival in patients after renal or rectal cancer surgery [13, 14]. However, the opposite effects of intraoperative hypertension were also reported. For example, Monk and co-workers [9] reported that intraoperative hypertension did not affect 30-day mortality; and in the study of Levin and colleagues [28] *,* 30-day survival was higher in hypertensive patients not taking antihypertensive medication. In the present study, patients with intraoperative hypertension had higher overall survival when compared with those without. It should be noted that our threshold of intraoperative hypertension is much lower than in many other studies [9, 13, 26] and is actually the upper normal limit. Considering that about one fourth of our patients had preoperative hypertension and more than half of our patients had “baseline” SBP of higher than 140 mmHg, an intraoperative SBP of 140 mmHg meant that it was closer to baseline level. This partially explains why our results showed favorable effects of intraoperative hypertension [29].

Importantly, the harmful effects of intraoperative hypotension have often been reported including that a previous elegant study reported “triple low” profoundly affected perioperative outcome [30]. Indeed, intraoperative hypotension is associated with an increased risk of myocardial injury, acute kidney injury, and even 30-day mortality [8, 9, 28, 31]. Furthermore, it seems that these harmful effects last long time after surgery. For example, Bijker and co-workers [11] reported that intraoperative hypotension was a predictor of 1-year mortality in the elderly after noncardiac surgery. In the study reported by Younes, Rogatko and Brennan [12], intraoperative hypotension episodes were associated with early recurrence in patients after surgery for liver metastases from colorectal origin. In line with the above findings, our results showed that, in patients undergoing lung cancer surgery, even a short duration of mild intraoperative hypotension might worsen long-term survival (Fig. 2, Tables 3 and 4).

The underlying mechanisms for how intraoperative hypotension impact long-term survival remain unclear but may include the following. First, intraoperative hypotension increases the risk of perioperative vital organ injury including myocardial injury and acute kidney injury [8, 31], each of which is associated with worsened overall survival [32, 33]. However, this was not the case in our patients as 94.7% (249/263) of patient deaths were caused by cancer. Second, microenvironmental hypoxia, which is a common feature in solid cancer [34], might have been aggravated by intraoperative hypotension and thus promoted cancer aggressiveness and metastasis via hypoxia inducible factor mechanisms [35, 36]. Third, hypoxia resulting from intraoperative hypotension might have augmented systematic inflammation [37] which enhances cancer recurrence and cancer-related death [38]. Interestingly, a recent study showed that individualized intraoperative blood pressure management reduced systemic inflammatory response syndrome and organ dysfunction after surgery [29] although its long-term impact on surgical outcome has not been reported yet.

The main strength of this study was the long-term follow-up which was completed by specialized personnel according to a standard procedure in a sufficient size patient population. Secondly, intraoperative hypo- and hypertension were defined according to their impacts on long-term survival after adjustment for confounding factors. Finally, intraoperative hypotension or hypertension alone or both in combination were analyzed separately.

Apart from the observational single-center nature, there are still some other limitations in the present study. We adopted binary definitions of intraoperative hypo- and hypertension and did not analyze the effects of duration of hypo−/hypertension. The precise data regarding pre- and intraoperative antihypertensive therapy were not collected because of lacking information in most of patient’s records; although previous studies revealed no associations between antihypertensive drugs [39], including the debatable β-blockers [40], with the risk of cancer mortality. We did not consider the influence of postoperative blood pressure as these data were not documented in the electronic medical record system; but the usual case is that blood pressure returned to baseline after awaking from anesthesia at the end of surgery. This is a further analysis of the database from a similar patient cohort, which might influence the discriminatory power for the selected outcome. Nonetheless, our results provide clues for further interventional studies.

## Conclusions

Our results showed that the estimated five-year overall survival rate following lung cancer surgery was the lowest (41.2%) in patients with only hypotension episodes during anesthesia and surgery, in comparison to those with only hypertension (66.5%), with both hyper- and hypotension (61.3%), and with neither hyper- nor hypotension (56.5%). After adjustment for confounding factors, intraoperative hypotension, but not hypertension, was associated with shortened overall survival. Interventional studies are needed to clarify the impact of intraoperative blood pressure management on long-term survival in lung cancer patients undergoing surgery.

## Supplementary information

**Additional file 1 : Table S1.** Baseline data and perioperative management.

**Additional file 2 : Table S2.** Data of postoperative follow-up and outcomes.

## Data Availability

The datasets used and analyzed in the current study are available from the corresponding author upon reasonable request.

## Abbreviations

SBP: Systolic blood pressure
HR: Hazard ratio
CI: Confidence interval
BMI: Body mass index
ASA: American Society of Anesthesiologists
TNM stage: Tumor-node-metastasis stage
IQR: Interquartile range
NSAIDs: Non-steroidal anti-inflammatory drugs
