# Supplementary material for: Intraoperative hypotension is associated with shortened overall survival after lung cancer surgery
Source: BMC Anesthesiol. 2020 Jun 29;20:160. doi: 10.1186/s12871-020-01062-2 (PMC7322881; doi:10.1186/s12871-020-01062-2)
Supplement: Supplementary file 1 — Additional file 1 : Table S1. Baseline data and perioperative management. [file 12871_2020_1062_MOESM1_ESM.docx]

**Table S1.** Baseline data and perioperative management

|  | Intraoperative hypertension (+), hypotension (-) (n = 167) ^a^ | Intraoperative hypertension (+), hypotension (+) (n = 119) ^a^ | Intraoperative hypertension (-), hypotension (-) (n = 69) ^a^ | Intraoperative hypertension (-), hypotension (+) (n = 160) ^a^ | p value |
| --- | --- | --- | --- | --- | --- |
| Age (yr.) | 63 (56-69) | 64 (58-69) | 57 (48-67) | 58 (49-65) | <0.001^b^ |
| Body mass index (kg m ^-2^) | 24.4 (22.8-26.8) | 24.4 (22.6-26.2) | 24.1 (22.3-25.8) | 23.4 (21.3-25.4) | 0.001^c^ |
| Male gender | 98 (58.7%) | 82 (68.9%) | 42 (60.9%) | 106 (66.3%) | 0.272 |
| Chronic smoking ^d^ | 90 (53.9%) | 64 (53.8%) | 35 (50.7%) | 88 (55.0%) | 0.949 |
| Preoperative comorbidity |  |  |  |  |  |
| Coronary heart disease | 18 (10.8%) | 8 (6.7%) | 0 (0.0%) | 4 (2.5%) | 0.001^e^ |
| Hypertension | 68 (40.7%) | 37 (31.1%) | 10 (14.5%) | 20 (12.5%) | <0.001^f^ |
| Diabetes mellitus | 20 (12.0%) | 23 (19.3%) | 4 (5.8%) | 9 (5.6%) | 0.002^g^ |
| Stroke | 10 (1.9%) | 4 (3.4%) | 1 (0.2%) | 2 (0.4%) | 0.098 |
| Preoperative chemotherapy | 16 (9.6%) | 10 (8.4%) | 12 (17.4%) | 19 (11.9%) [1] | 0.245 |
| Charlson Comorbidity Index | 0 (0-1) | 0 (0-1) | 0 (0-0) | 0 (0-0) | <0.001^h^ |
| ASA classification |  |  |  |  | <0.001^i^ |
| I | 36 (21.6%) | 38 (31.9%) | 32 (46.4%) | 77 (48.1%) |  |
| II | 121 (72.5%) | 74 (62.2%) | 37 (53.6%) | 81 (50.6%) |  |
| III | 10 (6.0%) | 7 (5.9%) | 0 (0.0%) | 2 (1.3%) |  |
| Pre-anesthesia SBP (mmHg) ^j^ |  |  |  |  | <0.001^k^ |
| <120 | 8 (4.8%) | 12 (10.1%) | 21 (30.4%) | 44 (27.5%) |  |
| 120-139 | 29 (17.4%) | 27 (22.7%) | 25 (36.2%) | 72 (45.0%) |  |
| 140-159 | 61 (36.5%) | 47 (39.5%) | 18(26.1%) | 32 (20.0%) |  |
| ≥160 | 69 (14.3%) | 33 (27.7%) | 5 (7.2%) | 12 (7.5%) |  |
| Type of anesthesia |  |  |  |  | <0.001^l^ |
| General | 143 (85.6%) | 81 (68.1%) | 66 (95.7%) | 115 (71.9%) |  |
| Combined epidural-general | 24 (14.4%) | 38 (31.9%) | 3 (4.3%) | 45 (28.1%) |  |
| Use of general anesthetics |  |  |  |  |  |
| Propofol | 147 (88.0%) | 109 (91.6%) | 61 (88.4%) | 142 (88.8%) | 0.797 |
| Dose of propofol (mg) ^m^ | 100 (100-150) | 100 (80-130) | 120 (80-145) | 105 (80-140) | 0.889 |
| Etomidate | 46 (27.5%) | 26 (21.8%) | 13 (18.8%) | 22 (20.6%) | 0.358 |
| Dose of etomidate (mg) ^m^ | 20 (16-20) | 20 (20-20) | 20 (18-30) | 20 (20-25) | 0.525 |
| Volatile anesthetics | 163 (97.6%) | 117 (98.3%) | 69 (100.0%) | 159 (99.4%) | 0.388 |
| Sevoflurane | 94 (56.3%) | 69 (58.0%) | 46 (66.7%) | 101 (63.1%) | 0.378 |
| Isoflurane | 69 (41.3%) | 48 (40.3%) | 23 (33.3%) | 58 (36.3%) | 0.604 |
| Nitrous oxide (N_2_O:O_2_=1-2:1) | 36 (21.6%) | 21 (17.6%) | 9 (13.0%) | 28 (17.5%) | 0.463 |
| Intraoperative crystalloid (ml) | 1350 (1100-1600) | 1500 (1100-2000) | 1400 (1100-1750) | 1250 (1100-1600) | 0.065 |
| Intraoperative artificial colloid (ml) | 500 (500-1000) | 500 (500-1000) | 500 (500-1000) | 500 (500-1000) | 0.246 |
| Estimation of blood loss (mL) | 200 (100-200) | 200 (100-200) | 200 (100-200) | 200 (100-200) | 0.660 |
| Intraoperative blood transfusion | 2 (1.2%) | 4 (3.4%) | 0 (0.0%) | 3 (1.9%) | 0.401 |
| Intraoperative vasoactive drugs | 15 (9.0%) | 38 (31.9%) | 7 (10.1%) | 41 (25.6%) | <0.001^n^ |
| Ephedrine | 11 (6.6%) | 35 (29.4%) | 6 (8.7%) | 37 (23.1%) | <0.001^o^ |
| Phenylephrine | 4 (2.4%) | 3 (2.5%) | 1 (1.4%) | 3 (1.9%) | 0.951 |
| Dopamine/norepinephrine | 1 (0.6%) | 0 (0.0%) | 0 (0.0%) | 1 (0.6%) | 0.764 |
| Perioperative opioids ^p^ | 167 (100.0%) | 118 (99.2%) | 68 (98.6%) | 159 (99.4%) | 0.569 |
| Dose of sufentanil equivalent (μg) ^p^ | 310 (240-320) | 270 (30-320) | 310 (225-332.5) | 245 (20-320) | <0.001^q^ |
| Perioperative flurbiprofen axetil | 111 (66.5%) | 63 (52.9%) | 48 (69.6%) | 89 (55.6%) | 0.026^r^ |
| Dose of flurbiprofen axetil (mg) ^m^ | 200 (200-300) | 200 (150-300) | 200 (150-200) | 200(200-200) | 0.198 |
| Perioperative dexamethasone | 81 (48.5%) | 71 (59.7%) | 35 (50.7%) | 79 (49.4%) | 0.253 |
| Dose of dexamethasone (mg) ^m^ | 10 (10-10) | 10 (10-10) | 10 (10-10) | 10 (10-10) | 0.470 |
| Duration of surgery (hr.) | 4 (3-5) | 4 (3.4) | 4 (3-4) | 4 (3-5) | 0.192 |
| Type of surgery ^s^ | [4] | [1] | [1] | [1] | 0.517 |
| Lobectomy | 119 (73.0%) | 90 (76.3%) | 45 (66.2%) | 120 (75.5%) |  |
| Pneumonectomy | 4 (2.5%) | 3 (2.5%) | 6 (8.8%) | 6 (3.8%) |  |
| Wedge resection | 15 (9.2%) | 10 (8.5%) | 5 (7.4%) | 13 (8.2%) |  |
| Bronchial resection | 6 (3.7%) | 7 (5.9%) | 4 (5.9%) | 10 (6.3%) |  |
| Conservative resection or biopsy | 19 (11.7%) | 8 (6.8%) | 8 (11.8%) | 10 (6.3%) |  |
| Mediastinal lymph node dissection | 146 (87.4) | 108 (90.8%) | 60 (87.0%) | 147 (91.9%) | 0.493 |

Values are median (interquartile range) or number (%). Numbers in square brackets indicate patients with missing data.

Abbreviations: ASA=American Society of Anesthesiologists, SBP=systolic blood pressure; p values were obtained using Kruskal-Wallis tests (for non-normally distributed continuous variables and ordinal variables), or Chi-square or Fisher’s exact test (for categorical variables).

^a^ Intraoperative hypertension was defined as a systolic blood pressure >140 mmHg for ≥5 min. Intraoperative hypotension was defined as a systolic blood pressure <100 mmHg for ≥5 min.

^b^ Compared with only hypertension, p=0.535 for both hyper- and hypotension, p=0.001 for neither hyper- nor hypotension, p<0.001 for only hypotension. p<0.017 was considered statistically significant after Bonferroni correction; same for the following post hoc multiple comparisons.

^c^ Compared with only hypertension, p=0.477 for both hyper- and hypotension, p=0.144 for neither hyper- nor hypotension, p=0.001 for only hypotension.

^d^ Smoking of half a pack of cigarettes per day for at least 2 years, either former or current smoker.

^e^ Compared with only hypertension, p=0.240 for both hyper- and hypotension, p=0.002 for neither hyper- nor hypotension, p=0.003 for only hypotension.

^f^ Compared with only hypertension, p=0.096 for both hyper- and hypotension, p<0.001 for neither hyper- nor hypotension, p<0.001 for only hypotension.

^g^ Compared with only hypertension, p=0.086 for both hyper- and hypotension, p=0.235 for neither hyper- nor hypotension, p=0.043 for only hypotension.

^h^ Compared with only hypertension, p>0.999 for both hyper- and hypotension, p=0.020 for neither hyper- nor hypotension, p=0.123 for only hypotension.

^i^ Compared with only hypertension, p=0.090 for both hyper- and hypotension, p<0.001 for neither hyper- nor hypotension, p<0.001 for only hypotension.

^j^ The last systolic blood pressure reading in the operating room before anesthesia induction.

^k^ Compared with only hypertension, p=0.016 for both hyper- and hypotension, p<0.001 for neither hyper- nor hypotension, p<0.001 for only hypotension.

^l^ Compared with only hypertension, p<0.001 for both hyper- and hypotension, p=0.028 for neither hyper- nor hypotension, p=0.002 for only hypotension.

^m^ Dosage among patients who had received the drug.

^n^ Compared with only hypertension, p<0.001 for both hyper- and hypotension, p=0.780 for neither hyper- nor hypotension, p<0.001 for only hypotension.

^o^ Compared with only hypertension, p<0.001 for both hyper- and hypotension, p=0.585 for neither hyper- nor hypotension, p<0.001 for only hypotension.

^p^ Including opioid consumption during anesthesia (fentanyl and/or sufentanil) and for postoperative analgesia (morphine and/or sufentanil), converted to intravenous sufentanil equivalent.

^q^ Compared with only hypertension, p=0.014 for both hyper- and hypotension, p>0.999 for neither hyper- nor hypotension, p<0.001 for only hypotension.

^r^ Compared with only hypertension, p=0.021 for both hyper- and hypotension, p=0.644 for neither hyper- nor hypotension, p=0.044 for only hypotension.

^s^ Conservative resection or biopsy was performed in patients with nonresectable cancer.
