# Supplementary material for: Intraoperative hypotension is associated with shortened overall survival after lung cancer surgery
Source: BMC Anesthesiol. 2020 Jun 29;20:160. doi: 10.1186/s12871-020-01062-2 (PMC7322881; doi:10.1186/s12871-020-01062-2)
Supplement: Supplementary file 2 — Additional file 2 : Table S2. Data of postoperative follow-up and outcomes. [file 12871_2020_1062_MOESM2_ESM.docx]

**Table S2.** Data of postoperative follow-up and outcomes

|  | Intraoperative hypertension (+), hypotension (-) (n = 167) ^a^ | Intraoperative hypertension (+), hypotension (+) (n = 119) ^a^ | Intraoperative hypertension (-), hypotension (-) (n = 69) ^a^ | Intraoperative hypertension (-), hypotension (+)  (n = 160) ^a^ | p value |
| --- | --- | --- | --- | --- | --- |
| Non-small cell lung cancer | 163 (97.6%) | 115 (96.6%) | 68 (98.6%) | 153 (95.6%) | 0.614 |
| Maximal tumor size (cm) | 3.0 (2.0-4.0) | 3.0 (2.0-4.0) | 3.0 (2.0-4.0) | 3.0 (2.0-4.0) |  |
| Tumor differentiation |  |  |  |  | 0.690 |
| Highly differentiated | 21 (12.6%) | 14 (11.8%) | 9 (13.0%) | 18 (11.3%) |  |
| Moderately differentiated | 104 (62.3%) | 73 (61.3%) | 44 (63.8%) | 94 (58.8%) |  |
| Poorly differentiated | 20 (12.0%) | 9 (7.6%) | 8 (11.6%) | 23 (14.4%) |  |
| Undifferentiated | 22 (13.2%) | 23 (19.3%) | 8 (11.6%) | 25 (15.6%) |  |
| Pathological TNM stage ^b^ | [12] | [4] | [3] | [12] | 0.047^c^ |
| I | 74 (47.7%) | 55 (47.8%) | 22 (33.3%) | 50 (33.8%) |  |
| II | 28 (18.1%) | 20 (17.4%) | 14 (21.2%) | 38 (25.7%) |  |
| III | 39 (25.2%) | 34 (29.6%) | 21 (31.8%) | 46 (31.1%) |  |
| IV | 14 (9.0%) | 6 (5.2%) | 9 (13.6%) | 14 (9.5%) |  |
| Occurrence of postoperative complications | 111 (66.5%) | 87 (73.1%) | 49 (71.0%) | 122 (76.3%) | 0.260 |
| Pneumothorax ^d^ | 100 (59.9%) | 77 (64.7%) | 42 (60.9%) | 106 (66.3%) | 0.636 |
| Pleural effusion ^e^ | 41 (24.6%) | 40 (33.6%) | 21 (30.4%) | 63 (39.4%) | 0.037^f^ |
| New-onset atrial fibrillation ^g^ | 17 (10.2%) | 11 (9.2%) | 10 (14.5%) | 15 (9.4%) | 0.667 |
| Atelectasis ^h^ | 10 (6.0%) | 3 (2.5%) | 4 (5.8%) | 8 (5.0%) | 0.571 |
| Surgical bleeding ^i^ | 5 (3.0%) | 1 (0.8%) | 1 (1.4%) | 5 (3.1%) | 0.539 |
| Pneumonia ^j^ | 1 (0.6%) | 2 (1.7%) | 0 (0.0%) | 1 (0.6%) | 0.590 |
| Pulmonary embolism ^k^ | 1 (0.6%) | 0 (0.0%) | 0 (0.0%) | 2 (1.3%) | 0.507 |
| Acute myocardial infarction ^l^ | 1 (0.6%) | 0 (0.0%) | 0 (0.0%) | 0 (0.0%) | 0.521 |
| Stroke ^m^ | 0 (0.0%) | 1 (0.8%) | 0 (0.0%) | 0 (0.0%) | 0.402 |
| Deep vein thrombosis ^n^ | 0 (0.0%) | 1 (0.8%) | 0 (0.0%) | 0 (0.0%) | 0.402 |
| Postoperative follow-up interval (yr.) | 5.4 (2.8-6.5) | 5.9 (2.1-7.1) | 5.2 (2.3-6.4) | 3.1 (1.2-6.3) | 0.004^o^ |
| Postoperative chemotherapy | 75 (44.9%) | 56 (49.6%) | 41 (59.4%) | 85 (53.1%) | 0.190 |
| Postoperative radiotherapy | 5 (3.0%) | 9 (7.6%) | 3 (4.3%) | 6 (3.8%) | 0.326 |
| Death during postoperative follow-up | 73 (43.7%) | 57 (47.9%) | 34 (49.3%) | 99 (61.9%) | 0.009^p^ |
| Cancer-specific death | 68 (40.7%) | 52 (43.7%) | 33 (47.8%) | 96 (60.0%) | 0.003^q^ |

Values are number (%) or median (interquartile range). Numbers in square brackets indicate patients with missing data.

Abbreviations: TNM=Tumor-Node-Metastasis; p values were obtained using Kruskal-Wallis tests (for non-normally distributed continuous variables and ordinal variables), or Chi-square or Fisher’s exact tests (for categorical variables).

^a^ Intraoperative hypertension was defined as a systolic blood pressure >140 mmHg for ≥5 min. Intraoperative hypotension was defined as a systolic blood pressure <100 mmHg for ≥5 min.

^b^ According to the 7th edition of the American Joint Committee on Cancer staging system.

^c^ Compared with only hypertension, p=0.858 for both hyper- and hypotension, p=0.043 for neither hyper- nor hypotension, p=0.056 for only hypotension. p<0.017 was considered statistically significant after Bonferroni correction; same for the following post hoc multiple comparisons.

^d^ Confirmed by chest X-ray or CT scan and required chest tube placement.

^e^ Confirmed by chest X-ray and required thoracentesis or chest tube drainage.

^f^ Compared with only hypertension, p=0.094 for both hyper- and hypotension, p=0.350 for neither hyper- nor hypotension, p=0.004 for only hypotension.

^g^ Confirmed by electrocardiographic monitoring and required pharmacological treatment.

^h^ Confirmed by chest x-rays, with desaturation, and necessitated physiotherapy and/or bronchoscopy.

^i^ Required second surgery for hemostasis.

^j^ New infiltration on chest X-ray with fever and treated with intravenous antibiotics.

^k^ Confirmed by computed tomographic pulmonary angiography and treated with anticoagulants.

^l^ Confirmed by clinical symptoms, electrocardiographic changes and elevated cardiac troponin I levels.

^m^ New focal neurologic deficit of more than 24 hours and confirmed by neurologic imaging.

^n^ Confirmed by lower limbs venous ultrasonography and treated with anticoagulants.

^o^ Compared with only hypertension, p=0.706 for both hyper- and hypotension, p=0.446 for neither hyper- nor hypotension, p=0.002 for only hypotension.

^p^ Compared with only hypertension, p=0.483 for both hyper- and hypotension, p=0.435 for neither hyper- nor hypotension, p=0.001 for only hypotension.

^q^ Compared with only hypertension, p=0.615 for both hyper- and hypotension, p=0.315 for neither hyper- nor hypotension, p< 0.001 for only hypotension.
